# Reproductive factors are crucial in the aetiology of breast cancer

**DOI:** 10.1054/bjoc.2000.1241

**Published:** 2000-06-02

**Authors:** S A Khuder, A B Mutgi

**Affiliations:** Medical College of Ohio, USA


*British Journal of Cancer* (2000) **83**(1), 133–134
© 2000 Cancer Research Campaign
doi: 10.1054/ bjoc.2000.1241, available online at http://www.idealibrary.com on IDEAL®

Letter to the Editor

### Reproductive factors are crucial in the aetiology of breast cancer

**Sir,**

Stewart et al (2000) propose that humans acquire mouse mammary tumour virus (MMTV) from mice. They advocate a viral aetiology for the high incidence of human breast cancer (HBC) and argue against other possible aetiological factors. However, their statistical analysis is simplistic and ignores social, cultural and demographic variables that are known to affect the risk of breast cancer (Gilliland, 1997). Two countries, Algeria (low incidence rate and in the lands of *M. domesticus*) and Finland (high incidence rate and in the lands of *M. musculus*) contradict the viral theory for HBC. While the authors do not explain the findings for Algeria they suggest cross-breeding between *M. domesticus* and *M. musculus* in Finland. We believe that socio-cultural and demographic variables contribute significantly to the low incidence. We also note that in Finland, HBC was most common in high social classes throughout the period 1971–1995 (Pukkala and Weiderpass, 1999).

It is well established that a woman's reproductive history influences her risk of HBC (Kelsey et al, 1993). Among reproductive and hormonal factors, the most important known determinants of breast cancer are late age at first birth and nulliparity, early menopause and use of hormone-replacement therapy. In Italy (a land of *M. domesticus*), the combination of risks associated with a high level of education, old age at first birth and nulliparity and older age at menopause accounted for 51% of breast cancer cases (Tavani et al, 1997). The lowest HBC incidence rate in the world (South Korea, a land inhabited by other mice) was attributed to late age at menarche, early age at natural menopause, early age at first full-term pregnancy and larger number of full-term pregnancies (Suh et al, 1996).

In Taiwan (a land inhabited by other mice) Chinese women were found to have lower incidence rates than white women of the same area (Chie et al, 1995). A substantial increase in HBC risk in women who migrated from Asia to the USA was demonstrated, with the risk doubling during the first decade after migration. Increased use of contraceptive soon after migration to the USA could possibly explain this rapid rise in risk (Ursin et al, 1995). In the USA (a high-incidence country) the incidence rates differed among 25 counties in the San Francisco Bay area and correlated with the distribution of known risk factors (Robbins et al, 1997; Prehn and West, 1998). Moreover, Hispanic women living in the USA have been shown to have the lowest incidence across most geographic regions of the USA (Jones et al, 1997).

The incidence of breast cancer is increasing more rapidly in societies that enjoyed a low incidence of the disease, such as most African countries. This is partly a result of the changing demographic profile, acquisition of 'Western' lifestyle, and the changing socioeconomic profile of the country (Adebamowo and Adekunle, 1999).

The reported relation between *M. domesticus* and HBC suffers from ecological bias because it does not take into consideration the density of mice population and its correlation with HBC incidence-rate. It will be interesting to see whether this relation will hold after adjusting for both human reproductive factors and mice population density.

*SA Khuder, AB Mutgi*
*Medical College of Ohio, USA*

### REFERENCES

Adebamowo CA and Adekule OO (1999) Case-controlled study of the epidemiological risk factors for breast cancer in Nigeria. *Br J Surg* **86**: 665–668

Chie WC, Chen CF, Chen CJ, Chang CL, Liaw YP and Lin RS (1995) Geographic variation of breast cancer in Taiwan: international and migrant comparison. *Anticancer Res* **15**: 2745–2749

Gilliland FD (1997) Ethnic differences in cancer incidence: a marker for inherited susceptibility? *Environ Health Perspect* **105** (suppl 4): 497–900

Jones LA, Gonzalez R, Pillow PC, Gomez-Garza SA, Foreman CJ, Chilton JA, Linares A, Yick J, Badrei M and Hajek RA (1997) Dietary fiber, Hispanics, and breast cancer risk? *Ann NY Acad Sci* **837**: 254–236

Kelsey JL, Gammon MD and John EM (1993) Reproductive factors and breast cancer. *Epidemiol Rev* **15**: 36–47

Prehn AW and West DW (1998) Evaluating local differences in breast cancer incidence rates: a census-based methodology (United States). *Cancer Causes Control* **9**: 511–517

Pukkala E and Weiderpass E (1999) Time trends in socio-economic differences in incidence rates of cancers of the breast and female genital organs (Finland, 1971–1995). *Int J Cancer* **31**: 56–61

Robbins AS, Brescianini S and Kelsey JL (1997) Regional differences in known risk factors and higher incidence of breast cancer in San Francisco. *J Natl Cancer Inst* **89**: 960–965

Stewart THM, Sage RD, Stewart AFR and Cameron DW (2000) Breast cancer incidence highest in the range of one species of house mouse, *Mus domesticus. Br J Cancer* **82**: 446–451

Suh JS, Yoo KY, Kwon OJ, Yun IJ, Han SH, Noh DY and Choe KJ (1996) Menstrual and reproductive factors related to the risk of breast cancer in Korea. Ovarian hormone effect on breast cancer. *J Korean Med Sci* **11**: 501–508

Tavani A, Braga C, La Vecchia C, Negri E, Russo A and Franceschi S (1997) Attributable risks for breast cancer in Italy: education, family history and reproductive and hormonal factors. *Int J Cancer* **70**: 159–163

Ursin G, Wu AH, Hoover RN, West DW, Nomura AM, Kolonel LN and Ziegler RG (1999) Breast cancer and oral contraceptive use in Asian-American women. *Am J Epidemiol* **150**: 561–567

doi: 10.1054/ bjoc.2000.1312, available online at http://www.idealibrary.com on IDEAL®

### Reproductive factors are crucial in the aetiology of breast cancer – a reply

**Sir,**

The letter by Professor Khuder raises several points of potential importance. Our statistical analysis correlating *Mus* species and human breast cancer (HBC) incidence is described as simplistic, ignoring social, cultural and demographic variables. Thus, it may suffer from ecological bias, due to the effect of hormonal promoters on the development of HBC. The greatest influence would likely be associated with fecundity, which is best reflected in the world statistics on ‘total fertility rate’ (TFR) (US Bureau of the Census, Report WP/98, World Population Profile (1998) US Government Printing Office: Washington DC, 1999).

TFR was evaluated as a potential confounder of the association of *M. domesticus* geography with human breast cancer incidence. For our sample of 39 countries (less two regions, Hawaii and ‘circumpolar Inuit’ for want of data), we analysed the reported 1990 (or 1998, where lacking) TFR for correlation with the world age-standardized incidence rate (WASIR) for female breast cancer (as in Stewart et al, 2000). The expected negative correlation of WASIR with TFR [$R = -0.327$, $P = 0.048$] was found. However, across Europe there was no difference in TFR between lands of *M. domesticus* and lands of other mice (mean TFR 1.656 ± SD 0.368, vs 1.657 ± 0.346, $P = 0.993$).

Internationally, excluding Europe, there was a higher reported TFR in *M. domesticus* lands (TFR 2.875 ± 0.822 vs 2.371 ± 0.971, $P = 0.244$). Overall, the crude difference in mean WASIR due to *M. domesticus* lands is +15.6, accounting for 38.3% of the observed variation in this sample. The TFR-adjusted difference in mean WASIR is + 17.4, accounting for 48.4% of variation, both highly statistically significant ($P < 0.001$). Thus, in addressing Professor Khuder’s concern about reproductive factors by adjusting for TFR, the association of WASIR with lands of *M. domesticus* was strengthened.

The report by McCredie et al (1999) on the incidence of HBC in Maori and non Maori women emphasizes that all parameters suggesting a lower incidence of HBC were seen in Maori women in a highly significant fashion, lower educational level, lower socio-economic status, lower age at first full term pregnancy, high parity and longer duration of breast-feeding. Despite this, the incidence of HBC in Maori women before the age of 54 is twice that of non-Maori women in New Zealand. Could this reflect a greater exposure of the Maori to *Mus domesticus* which occurs in both urban areas and native forests in New Zealand (King, 1982)?

In the paper by Chie et al (1995), no data on the incidence of HBC in white women is given in the text. White women form a minuscule proportion of the female population of Taiwan. The use of oral contraceptives in Asian women migrating to the USA, adjusted for age, ethnicity, study area, years since migration, family history of HBC and age at first full-term birth was not associated with increased risk of breast cancer (Ursin et al, 1999). The low incidence of HBC in Spanish women and Hispanic women living in the US is a fact. Genetic susceptibility to MMTV was not addressed in our paper, although it has been well studied in mice (Ross et al 1997; Golovkina, 2000).

In summary, adjustment of our analysis for a possible ecologic bias related to fecundity and hormonal influence on breast cancer increases the statistical significance of our reported association. We agree with Professor Khuder that one should seek a correlation in breast-cancer risk with more direct measures of contact and potential exposures to mice, such as local mouse population levels, or occupational exposures such as in farming (Khuder et al, 1998), or in laboratory work with experimental handling of mice (Dion et al, 1986). Some areas of the world do have wide fluctuations in *M. domesticus* population levels due to epizootic diseases, or climatic variations. One must keep in mind that the MMTV is the proposed cause, and that *M. domesticus* would be a surrogate of MMTV exposure. The actual risk will depend on the likely modes of MMTV transmission, exposure, and the burden of infectious MMTV in the resident mouse population.

*THM Stewart, CA Donnelly, RD Sage, DW Cameron, AFR Stewart*

### REFERENCES

Chie WC, Chen CF, Chen CJ, Chang CL, Liaw YP and Lin RS (1995) Geographic variation of breast cancer in Taiwan: international and migrant comparison. *Anticancer Res* **15**: 2745–2749

Golovkina TV (2000) A novel mechanism of resistance to mouse mammary tumor virus infection. *J Virol* **74**: 2752–2759

Dion AS, Girardi AJ, Williams CC and Pomenti AA (1986) Serologic responses to murine mammary tumor virus (MuMTV) in MuMTV-exposed laboratory personnel. *J Natl Cancer Inst* **76**: 611–619

Khuder SA, Schaub EA and Keller-Byrne JE (1998) Meta-analyses of non-Hodgkin’s lymphoma and farming. *Scand J Work Environ Health* **24**: 255–261

King CM (1982) Age structure and reproduction in feral New Zealand populations of the house mouse (*Mus musculus*), in relation to seedfall of southern beech. *New Zealand J Zool* **9***: 467–480

McCredie M Paul C, Skegg DC and Williams S (1999) Breast cancer in Maori and non-Maori women. *Int J Epidemiol* **28**: 189–195

Ross SR, Dzuris JL, Golovkina TV, Clemmons WC and van den Hoogen B (1997). Mouse mammary tumor virus (MMTV), a retrovirus that exploits the immune system. Genetics of susceptibility to MMTV infection. *Medicina (B Aires)* **57**: 34–42

Stewart TH, Sage RD, Stewart AF and Cameron DW (2000) Breast cancer incidence highest in the range of one species of house mouse, *Mus domesticus. Br J Cancer* **82**: 446–451

Ursin G Wu AH, Hoover RN, West DW, Nomura AM, Kolonel LN, Pike MC and Ziegler RG (1999) Breast cancer and oral contraceptive use in Asian-American women. *Am J Epidemiol* **150**: 561–567

 *© 2000 Cancer Research Campaign*

